# Exercise Intolerance That Resolved After venous Stenting of the Inferior Vena Cava

**DOI:** 10.1177/15385744231188801

**Published:** 2023-07-05

**Authors:** Jay M. Bakas, Adriaan Moelker, Wendy S. J. Malskat, Marie Josee E. Van Rijn

**Affiliations:** 1 Department of Surgery, 6993 Erasmus University Medical Center, Rotterdam, Netherlands; 2 Department of Radiology and Nuclear Medicine, 6993 Erasmus University Medical Center, Rotterdam, Netherlands; 3 Department of Dermatology, 6993 Erasmus University Medical Center, Rotterdam, Netherlands

**Keywords:** Cardiovascular abnormalities, venous thrombosis, endovascular procedures, hemodynamics

## Abstract

Venous stenting could alleviate exercise intolerance associated with chronic inferior vena cava (IVC) obstruction. We describe a 36-year-old male patient with an unknown IVC-obstruction. The obstruction was discovered after a bi-iliac deep vein thrombosis (DVT). The thrombus was resolved using thrombolysis. In the chronic phase, the patient developed exercise intolerance without any leg-specific symptoms or signs. Venous stenting was performed to open the IVC-obstruction, 1 year after the acute DVT. His physical condition improved, but cardiac magnetic resonance imaging at rest did not reveal hemodynamical changes after stenting. The Short Form Health Survey (SF-36) physical and mental component summaries were increased from 40.3 to 46.1 and 42.2 to 53.7, respectively. In patients with iliocaval obstruction, improved venous flow without changes in resting hemodynamics can enhance exercise intolerance and quality of life, even in the absence of leg symptoms. Diagnostic tools performed only at rest may miss abnormalities.

Partial or complete inferior vena cava (IVC) obstruction has a prevalence around 0.15%.^1^ It is often referred to as a congenital disorder, but may also result from thrombotic events in early childhood.^2^ Assessment of IVC-anomalies, including hypoplasia or aplasia is needed in DVT patients, especially those with iliofemoral DVT.^3^ Young patients with chronic IVC-obstruction may present asymptomatic, but are at risk for acute deep vein thrombosis (DVT), presenting at a mean age of twenty-five.^2^ Patients tend to develop post-thrombotic syndrome (PTS) later in life, which may cause pain, swelling, and/or ulceration of the leg.^4^ Exercise intolerance including dyspnea, exhaustion and turning pale during physical activities, is also observed.^2^

Deep venous stenting is an accepted treatment for symptomatic iliofemoral obstruction,^5^ but evidence is limited supporting its use for exercise intolerance. Endovenous recanalization, followed by venous stenting, could relieve exercise intolerance in IVC-obstruction.^6^ Cardiorespiratory functioning tests can be used to evaluate exercise intolerance in IVC-obstruction and its response to treatment.^7^ However, the impact of treatment on hemodynamics and quality of life (QoL) is unclear. Venous blood return is altered in IVC-obstruction, with the azygos vein, ascending lumbar veins, and abundant communicating veins becoming more prominent.^8^ Venous recanalization presumably resolves exercise intolerance by enhanced venous blood return.

This case-report presents a patient with exercise intolerance due to IVC-obstruction, which was accidentally discovered at presentation with an acute iliofemoral DVT. One year following acute DVT, the patient underwent venous stenting. Hemodynamics, clinical features, and quality of life (QoL) were evaluated pre- and post-procedure. Informed consent was obtained from the individual described in this case-report.

## Case Presentation

### Acute Phase

A 36-year-old male patient was referred to our academic hospital for endovenous treatment of acute bi-iliac DVT with coincidental finding of IVC-obstruction. One week earlier, he was admitted for iliocaval DVT and treated conservatively with a heparin pump elsewhere, subsequently replaced with a direct-oral-anticoagulant. He experienced minimal back pain and bilateral inguinal pain at discharge. However, he was readmitted after 3 days due to worsening of complaints.

**Figure 1.**
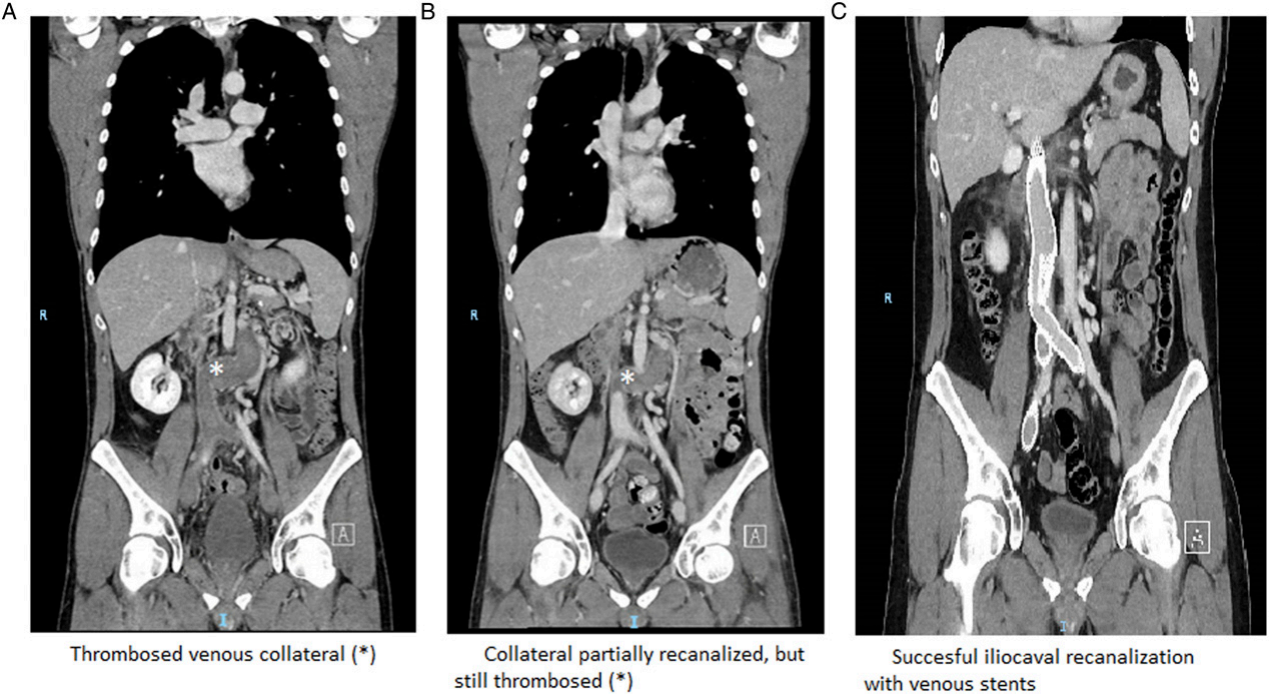
Computed tomography venography before thrombolysis (A); after thrombolysis (B); after venous stenting (C).

Upon arrival at our institution, the patient had a fever, malaise, back pain, severe bilateral groin pain, and impaired mobility. Notably, leg symptoms and/or signs were absent. Duplex ultrasound (DUS) and Computed tomography venography (CTV) revealed an extensive DVT from the femoral veins above the knee until the IVC, including a thrombosed collateral vein (Figure 1A). From the collateral vein upwards, the IVC demonstrated a small lumen, extending to the level of the hepatic veins.

Thrombolysis successfully restored patency of both iliac veins and the most distal segment of the IVC, but was stopped due to bleeding complications before the collateral opened (Figure 1B). Recanalization of the chronic IVC-obstruction was initially waived, because most symptoms had resolved.

### Chronic Phase

During the 6-month-control, the patient reported exercise intolerance. His complaints consisted of reduced physical condition, dyspnea, exhaustion and paleness during physical activities such as walking and cycling, without any leg-specific symptoms or signs. These complaints were absent prior to his acute DVT. Duplex ultrasound and CTV revealed a re-occlusion of the common iliac veins and distal IVC, with patent external iliac, femoral, and popliteal veins.

Venous stenting was planned to improve cardiac preload and to resolve complaints. Cardiac magnetic resonance imaging (MRI) and the Short Form Health Survey (SF-36) questionnaire were used to evaluate the effect, both pre- and post-procedure.

### Procedure

The first attempt of recanalization failed, but in a second attempt, the IVC was successfully stented. Both femoral veins and the right jugular vein were punctured for access. The IVC was recanalized after a snaring procedure creating a through and through wire. Intravascular ultrasound (IVUS) determined stent position and length after percutaneous transluminal angioplasty (24 mm for IVC, 16 mm for iliac veins). The IVC was stented right below the hepatic veins with 3 SinusXL (OptiMed®, Ettlingen, Germany) overlapping stents (80 × 24 mm + 80 × 24 mm + 16 × 24 mm), in which two BeYond (Bentley®, Hechingen, Germany) stents (16 × 150 mm) were placed in a kissing fashion extending into the external iliac veins, with one additional stent (16 × 120 mm) on the right side.

### Outpatient Clinic

Figure 1C shows patent venous stents without signs of complications. The patient received low-molecular-weight heparin and was discharged the next day. Anticoagulation was switched to a vitamin-K-antagonist after 2 weeks. Duplex ultrasound revealed patent venous stents until date (current follow-up 6 months). The patients’ physical condition improved, allowing him to exercise (daily activities, walking and cycling) without dyspnea, dizziness, or paleness. He also felt fitter during the day.

### Heart Volumes

Table 1 shows end-diastolic volume (EDV), end-systolic volume (ESV), systolic volume (SV), ejection fraction (EF), and 2D-flow measures of the cardiac valves. Heart volumes were similar before and after venous stenting at rest, and the forward stroke volume of the aortic, pulmonic, and atrioventricular valves aligned with these results.

**Table 1.** Cardiac Magnetic-Resonance Imaging (MRI), Including 2D Flow and Heart Volumes Before and After Deep Venous Stenting.

Left ventricle MRI measures	Before stenting	After stenting + 2 months
EDV (mL/m^2^)	99	94
ESV (mL/m^2^)	39	40
SV (mL/m^2^)	60	54
EF (%)	60	57
Right ventricle		
EDV (mL/m^2^)	100	96
ESV (mL/m^2^)	41	41
SV (mL/m^2^)	60	55
EF (%)	60	57
Forward stroke volume		
Aortic valve (mL/beat)	126	123
Mitral valve (mL/beat)	113	138
Pulmonic valve (mL/beat)	117	120
Tricuspidal valve (mL/beat)	106	120

LV: Left ventricle, RV: right ventricle; EDV: End-diastolic volume; ESV: End-systolic volume; SV: systolic volume; EF: ejection fraction.

### Quality of Life

Table 2 shows the SF-36 before and 3 months after venous stenting, with reference values of an age-adjusted Dutch cohort.^9^ The SF-36 is divided in physical functioning, role limitations due to physical health (role-physical), bodily pain, general health, vitality, social functioning, role limitation due to emotional problems (role-emotional), and mental health.^10^ Each domain scores between 0 and 100, with higher scores indicating a better QoL. Domains are summarized in a mental and physical component summary.

**Table 2.** Quality of Life Before and After Deep Venous Stenting.

SF-36 domains (0-100)	Before stenting	+3 months after stenting	Dutch population (35-44 years old)
Physical functioning	85.0	90.0	90.0
Role-physical	25.0	75.0	82.9
Bodily pain	74.0	74.0	83.8
General health	25.0	52.0	74.0
Vitality	50.0	70.0	67.1
Social functioning	50.0	75.0	88.0
Role-emotional	33.3	100.0	82.2
Mental health	80.0	80.0	76.9
Physical component summary	40.3	46.1	NR
Mental component summary	42.2	53.7	NR

A higher SF-36 score indicating a better quality of life (0-100); age-adjusted SF-36 outcomes of the general Dutch cohort were extracted from van der Zee et al (1993); NR = not reported.

All SF-36 domains remained stable or increased after venous stenting. Disabilities before the procedure were common in the role-physical, general health, and role-emotional domains with increased mean differences between 27.0 and 76.7 post-procedure. Physical and mental component summary also increased with 5.8 and 11.5 points, respectively.

## Discussion

We presented a young male patient with exercise intolerance due to iliocaval obstruction. Endovenous recanalization followed by venous stenting led to complete resolution of his symptoms 1 year after the initial DVT. His SF-36 scores increased and were comparable to those of an age-matched Dutch cohort. However, there were no changes in heart volumes at rest before and after venous stenting.

Exercise intolerance is often underestimated as a consequence of chronic (ilio)caval obstruction.^11^ It is not regularly evaluated in venous outflow obstruction.^12^ Currently, exercise intolerance also is not included in any PTS score. Inadequate return of venous blood to the heart may cause exercise intolerance. Reduced venous return from the lower limbs during exercise reduces stroke volume and cardiac output in iliocaval outflow obstruction.^11^ Venous stenting presumably resolves exercise intolerance, by enhancing venous return and increasing cardiac output.

Evaluating cardiac hemodynamics at rest may fail to detect venous abnormalities, as resting venous capacitance may not reveal the increased demands of venous volume during exercise.^11^ Despite unchanged pre- and post-venous stenting heart volumes, that were within the normal range, our patient experienced an improvement, as evidenced by SF-36 scores. Cardiac volumes were also normal at rest in patients with exercise intolerance after IVC-ligation, while lower volumes were measured during exercise.^13^ Therefore, diagnostic tools may miss abnormalities if only performed at rest.

Another lesson learned is that in acute DVT resulting from chronic IVC-obstruction, the IVC may be left untreated, with only fresh clot removal followed by lifelong treatment with anticoagulants. However, if collateral vessels remain occluded, persisting symptoms and signs may necessitate invasive treatment during follow-up.

## Conclusion

This case increases awareness for symptoms and signs related to physical exercise in venous iliocaval obstruction. Future studies are needed to reveal how venous stenting affects hemodynamics during exercise in IVC-obstruction. Understanding the impact of venous stents on cardiac hemodynamics and exercise-related complaints, may improve treatment selection for IVC-obstruction.
